# Biochemical Mechanisms of Genetic Recombination and DNA Repair

**DOI:** 10.1146/annurev-biochem-083024-113931

**Published:** 2025-03-28

**Authors:** Vivek B. Raina, Aidan Jessop, Eric C. Greene

**Affiliations:** Department of Biochemistry & Molecular Biophysics, Columbia University, New York, NY, USA

**Keywords:** homologous recombination, DNA end resection, homology search, crossover, noncrossover, meiotic recombination

## Abstract

Genetic recombination involves the exchange of genetic material between homologous sequences of DNA. It is employed during meiosis in sexually reproducing organisms or in somatic cells to accurately repair toxic DNA lesions like double-strand breaks and stalled replication forks. In these separate roles, recombination drives genetic diversity by enabling reshuffling of parental genetic information while also serving as a molecular safeguard against the deleterious effects of gross chromosomal rearrangements or mutagenic insults arising for either endogenous or exogenous reasons. In both cases, efficient recombination ensures faithful transmission of genetic information to subsequent generations. In this review, we provide an exploration of the biochemical mechanisms driving genetic recombination, elucidating the molecular intricacies of fundamental processes involved therein with a focus on mechanistic insights gained into these processes using biochemical and single-molecule techniques.

## 1. INTRODUCTION

Cellular genomic content is subjected to numerous insults arising because of environmental mutagens, radiation, or errors during DNA replication. In certain scenarios, however, cellular machinery is also programmed for the induction of DNA damage, for example, during meiosis, T-cell receptor formation, and antibody class switching in B lymphocytes (1–4). DNA lesions can manifest in different forms ranging from a single-base modification to double-strand breaks (DSBs). In the absence of precise and regulated repair, DNA lesions can lead to the accumulation of deleterious mutations or gross chromosomal aberrations that may result in disorders such as cancer (5, 6). To maintain genomic stability in the face of this incessant DNA damage, cells have evolved a range of repair pathways, each specialized to detect and repair a specific type of damage. To rectify small, non-helix-distorting lesions such as oxidative DNA damage and alkylated bases, cells primarily employ the base excision repair (BER) pathway (7–9). Nucleotide excision repair (NER) is initiated in response to DNA experiencing bulky, helix-distorting lesions such as UV-induced pyrimidine dimers and chemical adducts (10–14). For correcting errors that arise during DNA replication, cells employ the mismatch repair (MMR) pathway, which recognizes and repairs mispaired nucleotides and restores the correct base pairing (15–18). To repair DNA lesions in the form of DSBs, cells employ either homologous recombination (HR) or nonhomologous end joining (NHEJ) (19, 20). NHEJ operates throughout the cell cycle, whereas HR functions primarily during the S and G_2_ phases of the cell cycle. There are several comprehensive reviews detailing the processes involved in the BER, NER, MMR and NHEJ pathways (8, 9, 12–14, 17–20). In this review, we focus on HR-mediated repair of DSBs.

DSBs arise when the phosphate backbones of the two complementary DNA strands are broken simultaneously. DSBs are considered one of the most cytotoxic forms of DNA lesions because both strands of the DNA helix are broken, meaning that they have the potential for complete loss of genetic information, if not correctly repaired (5, 6). DSBs can occur as a result of damage inflicted by either exogenous or endogenous sources. Exogenous causes primarily include exposure to factors like ionizing radiation and certain chemical mutagens. Even in the absence of exogenous stress, DNA within cells frequently suffers damage arising from endogenous causes like oxidative stress or replication errors. Numerous cellular obstacles such as unusual chromatin structures or collisions between the replication machinery and transcription factors lead to collapse of the replication fork, which can result in DSBs. In addition to these DSBs, which are inadvertent in nature, cells have developed a programmed machinery to introduce DNA DSBs at specific junctures within their cell cycle (1–4). Despite the constant damage that a cellular DNA encounters, genomic changes are exceptionally rare, highlighting the remarkable efficiency of the repair pathways that function in response to these DNA DSB lesions. HR is one such highly accurate DNA repair pathway that repairs DSB lesions by using a homologous sequence found elsewhere in the genome, generally on a sister chromosome in somatic cells and a homologous chromosome in gametic cells, to restore the genetic information that is lost at the break site. The molecular players involved in HR recognize and process the DSB site to generate a single-stranded DNA (ssDNA) overhang that is used to pair with a complementary sequence on the homologous DNA. Using the intact homologous sequence as a template, DNA polymerase extends the broken strand, filling in the missing genetic information. After DNA synthesis, the repaired strands are rejoined, and the DNA structure is returned to its original configuration, restoring the continuity of the double helix. This multistep process requires a myriad of protein factors, with correct execution depending on the intricate coordination between the steps.

In this review, we discuss different steps involved in the process of HR and how they are regulated. We begin the discussion with the detection and processing of the DSB site, followed by the process of homology search and strand invasion and the molecular decisions that determine the choice of repair pathways within the context of HR. At the end, we discuss a specialized form of programmed DSB and its repair by HR that occurs during the meiotic cellular program. Overall, we highlight the insights gained into the roles played by different HR repair factors, with an emphasis on knowledge gained through studies in budding yeast and human cells (Table 1), and describe the regulatory biochemical mechanisms governing the eventual repair outcome.

## 2. GETTING READY: INITIATION OF HOMOLOGOUS RECOMBINATION BY DNA END RESECTION

**Figure 1:**
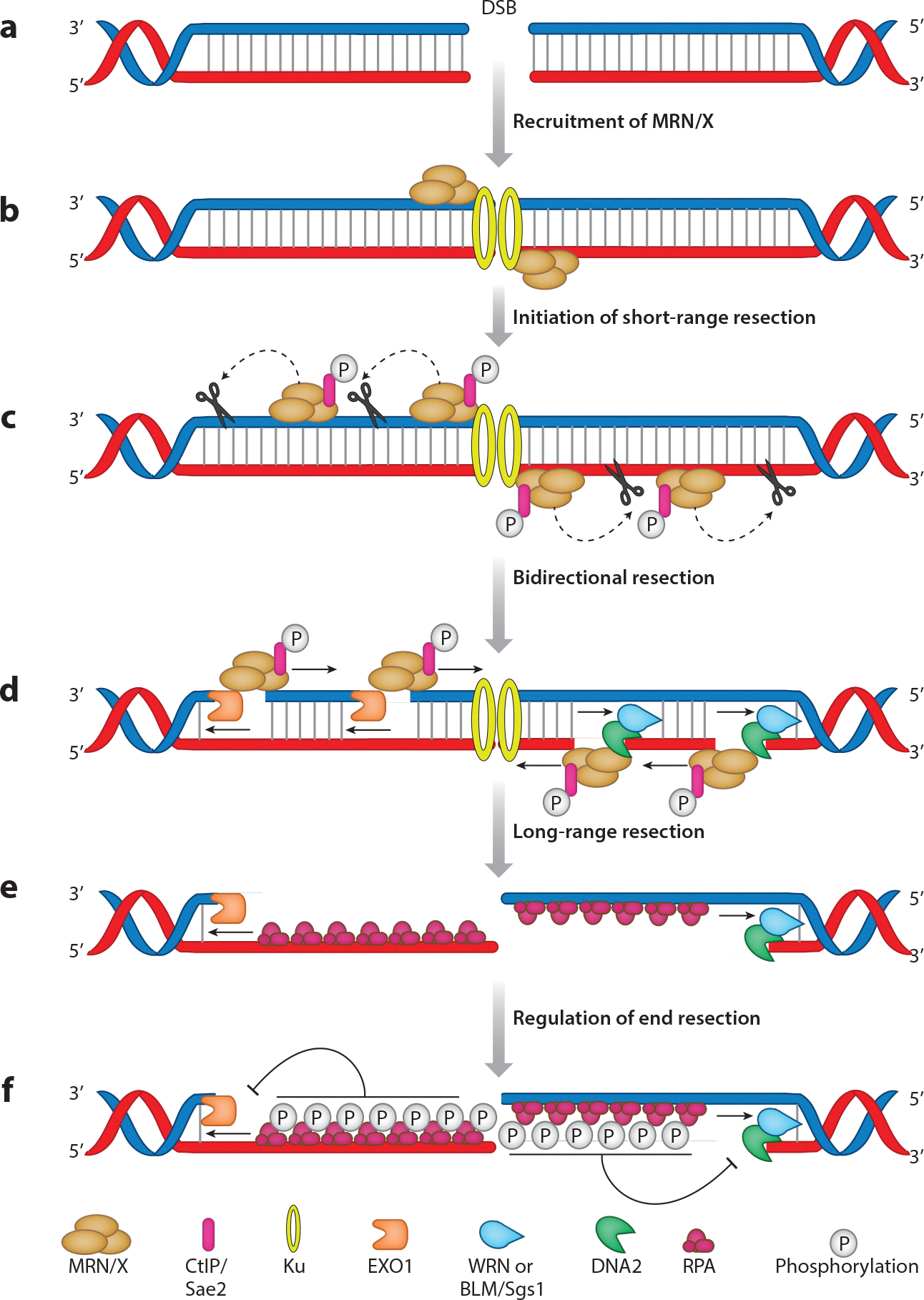
DNA end resection. (*a*) DSBs occur on the DNA. (*b*) Ku and MRN/X are recruited. (*c*) Phosphorylated CtIP–Sae2 is recruited by MRN/X and stimulates the nuclease activity of MRE11, leading to the introduction of DNA nicks and the initiation of short-range resection. (*d*) EXO1 or DNA2 plus BLM or WRN initiate bidirectional resection. (*e*) Long-range resection yields large stretches of single-stranded DNA that are rapidly bound by RPA. (*f*) RPA phosphorylation provides negative feedback to the end resection machinery. Abbreviations: BLM, Bloom helicase; CtIP, CtBP-interacting protein; DSB, double-strand break; EXO1, exonuclease I; MRN/X, MRE11–RAD50–NBS1/Xrs2 complex; RPA, replication protein A; WRN, Werner helicase.

DSBs need to be immediately recognized and processed. During processing, one strand of the DNA is nucleolytically cleaved in a process called DNA end resection (Figure 1). The machinery involved in DNA end resection operates under strict regulation to ensure that correct polarity of the nucleolytic degradation is maintained: Degradation occurs in the 5′-to-3′ direction from the ends, while the 3′ free end of the DNA is protected from degradation (Figure 1) (21–24). Protection of the 3′ overhang ensures that the genetic information is maintained, and it serves as the strand that invades the homologous DNA sequence during repair. DNA end resection is executed through the coordinated interplay of different proteins in a two-step manner: Short-range resection, in which one strand of DNA is clipped in near proximity to the DSB ends (25, 26), is followed by long-range resection, in which long stretches of 3′ ssDNA are generated (Figure 1) (23, 25, 27, 28). Long-range DNA end resection plays a pivotal role in ensuring that the high-fidelity HR pathway is employed for the repair of DSBs.

### 2.1. Short-Range End Resection

The MRN complex consists of three highly conserved proteins, MRE11, RAD50, and NBS1 (MRX for Mre11–Rad50–Xrs2 in *Saccharomyces cerevisiae*), and serves as the central component involved in sensing DSBs and executing short-range resection (29–32). In mammalian cells, the MRN complex is recruited to damaged sites through mechanistically distinct interactions of the NBS1 component with γ-H2AX (phosphorylated H2AX, a histone H2A variant) as well as with RAD17, a cell cycle checkpoint–regulating protein (33, 34). In vitro single-molecule experiments revealed that MRN can diffuse along a DNA strand (35). MRE11 is the main player in the MRN/X complex for both its DNA-binding and nucleolytic activities. Although MRE11 binds to linear and circular DNA with the same efficiency (36), there is a higher likelihood of DSB end occupancy by MRN if the ends are prebound by the NHEJ factors Ku70 and Ku80 (the Ku complex) (35). Yeast and mammalian MRN/X complexes form heterohexameric molecular assemblies. Recent cryo–electron microscopy (cryo-EM) studies of the MRN complex from *Chaetomium thermophilum*, combined with previous structural data, show that the MRN/X complex forms a dynamic molecular structure consisting of a globular head domain, two protruding coiled-coil arms extending up to 60 nm in length, and a distal dimerization zinc-hook domain at the apex of the structure (36–38). DNA binding is achieved through the globular head domain via two conserved DNA-binding units present in MRE11 (39). The protruding arms of the MRN complex also engage in interactions that are thought to play a role in bridging distal DNA elements (38, 40). Notably, detailed structures of the complete MRN/X complex have not yet been defined, but given the high level of interest, it seems inevitable that they will be solved, and their availability would be a major advance for the field.

As indicated above, MRE11 is responsible for the nucleolytic activity of the MRN/X complex. In vitro experiments using purified yeast Mre11 show that Mre11 degrades DNA with a 3′-to-5′ polarity (41), as opposed to the 5′-to-3′ polarity of the resection required to obtain 3′ ssDNA overhangs. This observation is reconciled by a model that divides short-range resection into a two-step process. First, a nick is created tens of nucleotides away from the DSB via the endonucleolytic activity of Mre11; growing evidence suggests that there is a stepwise increase in ssDNA formation by virtue of repetitive nicking (Figure 1c,d) (42, 43). Moreover, in yeast, it has been shown that the endonuclease activity of the MRX complex is governed by local DNA signatures with a specific preference for endonucleolytic activity 5′ of cytosines within AT-rich sequences (43, 44). At the site of endonucleolytic cleavage, Mre11 then starts to degrade the DNA in a 3′-to-5′ direction, moving toward the DSB site (Figure 1d). What protects the 3′ ends at the DSB sites from Mre11 activity? The Ku protein complex is proposed to rapidly bind the DSB sites and shield the 3′ ends from the exonuclease activity of Mre11 (45, 46). Single-molecule data suggest that the Ku protein is then removed by the activity of Mre11 to provide a clean 3′ overhang (35).

The other components of the MRN/X complex, RAD50 and NBS1/Xrs2, play a role in regulating the activity of MRE11. RAD50 regulates MRE11 nuclease activity through its ATP-binding and hydrolysis activities (47, 48). ATP hydrolysis causes a conformational change within the complex that leads to the availability of the DNA for MRE11’s nucleolytic activity. In mammalian systems, NBS1 is essential for the nuclear localization of both MRE11 and RAD50 and is important for MRE11’s nucleolytic activity (49, 50). Apart from RAD50 and NBS1, another factor that plays an important role in regulating the nucleolytic activity of MRE11 is CtIP, a CtBP-interacting protein (see Section 2.3) (51, 52).

### 2.2. Long-Range End Resection

Following the initial DNA cleavage events catalyzed by MRN–CtIP, long-range resection can then be initiated via the concerted effort of various helicases and nucleases (Figure 1e). Proteins responsible for long-range resection degrade DNA in the 5′-to-3′ direction and are introduced at the MRE11-mediated DNA cleavage sites, initiating what is commonly termed bidirectional resection (Figure 1d) (53). Long-range resection is catalyzed by two partially redundant pathways involving the EXO1 and DNA2 nucleases (Figure 1e). EXO1 can act alone to degrade one strand of the DNA (54). However, DNA2 can degrade only ssDNA, and hence, it functions in combination with RecQ helicases. The evolutionary rationale behind these seemingly redundant end resection pathways remains an open question. Notably, the EXO1- and DNA2-mediated pathways are only partially redundant, as indicated by genetic disorders associated with different end resection factors and consistent with the idea that they can each function in unique scenarios (28, 55). One possible explanation is that DNA2 and EXO1 may work on different DNA structures, although this idea remains to be proven.

EXO1 belongs to the Rad2/XPG family of metallonucleases (56). Other proteins that belong to this family of nucleases include FEN1 and GEN1, both of which are known to play roles in replication and repair (57). EXO1 was first described as an exonuclease that degrades double-stranded DNA (dsDNA) to leave long stretches of ssDNA, which are necessary for meiotic recombination in *Schizosaccharomyces pombe* (58). In human cells, EXO1 depletion leads to hypersensitivity to ionizing radiation and chromosomal instability, hallmarks of HR defects (59). Biochemical characterization of EXO1 revealed that EXO1 has a preference for DNA molecules with 3′ overhangs (54). The N-terminal domain of EXO1 coordinates two Mg^2+^ ions and binds to the DNA phosphate backbone (60). EXO1 exhibits 5′ flap activity and can bind to and process DNA gaps and nicks (61, 62). This ability of EXO1 to bind and process DNA nicks is employed at the nucleolytic site of MRN/X cleavage (49, 63).

DNA2 functions in parallel with EXO1, ensuring the formation of long stretches of ssDNA. DNA2 has nuclease, helicase, and ATPase domains and an Fe–S cluster (64). DNA2 was discovered in *S. cerevisiae* through the characterization of a temperature-sensitive and DNA replication–defective mutant strain (65). Biochemical characterization shows that DNA2 requires the divalent cations and an intact Fe–S cluster to support both its ATPase and nuclease activities; additionally, both ATP and Mg^2+^ modulate its helicase and nuclease activities (66, 67). DNA2 works in a processive manner in the presence of replication protein A (RPA), a single-strand-binding heterotrimeric complex (68). Overall, DNA2 has a cylindrical shape with a narrow central cavity large enough only for ssDNA to thread through with the 5′ end of DNA positioned toward the helicase domain and the 3′ end toward the nuclease domain (64). DNA2 functions in conjunction with helicase partners that unwind the DNA to provide the ssDNA as the substrate for its nuclease activity. These helicase partners for DNA2 are BLM (Sgs1 in yeast) and WRN (69–72). Both BLM and WRN belong to the RecQ family of helicases and function in a partially redundant manner (72).

### 2.3. Regulation of DNA End Resection

DNA end resection must be tightly regulated and coordinated with the cell cycle and DNA damage checkpoints (23, 73–75). End resection is suppressed during G_1_ but available during the G_2_ and S phases to increase the probability that repair occurs via a sister chromatid instead of a homolog, thus decreasing the chance of heterozygosity loss. Cyclin-dependent kinases (CDKs) play a major role in this regulation. The MRN complex, CtIP, DNA2, and EXO1 are all posttranslationally modified by CDKs. NBS1 is phosphorylated at serine 432 in a cell cycle–specific manner to stimulate end resection (76). CtIP is phosphorylated at serine 327 and threonine 847 during early S phase. CtIP phosphorylation positively regulates short-range resection by activating the MRN complex. During G_2_/M phase, CtIP is downregulated in a PLK1 phosphorylation–dependent manner (77, 78). CDKs directly phosphorylate EXO1 and DNA2, which promotes their recruitment to DNA lesions (79, 80). Dbf4-dependent kinase, another cell cycle–dependent kinase, is also responsible for stimulating DNA end resection through its kinase activity (81).

DNA end resection is mechanistically intertwined with the DNA damage response (DDR). Defects in DDR activation allow cells to progress further into the cell cycle with unrepaired DNA damage, which can then lead to genomic instability. Mammalian ATM (Tel1 in yeast) and ATR (Mec1 in yeast) are the two master regulators of DDR. ATM/Tel1 kinase is recruited to DSBs by the MRN/X complex, where it phosphorylates a myriad of substrates to promote DDR (82). After recruitment by MRN, ATM is activated by autophosphorylation (83). Upon activation, ATM in turn promotes CtIP recruitment to damage sites (84). After short-range resection, DDR signaling is switched from being driven primarily by ATM/Tel1 to being driven by ATR/Mec1. The ssDNA generated after initiation of resection is quickly bound by RPA; ATR/Mec1 is then recruited to the RPA–ssDNA complex via ATR/Mec1’s interaction with its obligate partner ATRIP/Ddc2 (85). RPA phosphorylation by ATM and ATR downregulates long-range resection by inhibiting the helicase activity of BLM (Figure 1f) (86). ATR-dependent phosphorylation and UBC9-mediated SUMOylation targets EXO1 for proteosomal degradation, thus downregulating EXO1-mediated resection (87, 88).

Additional factors such as HELB, BRCA1–BARD1, 53BP1, DYNLL1, the Shieldin complex, and CST also influence end resection (89–95). HELB is an RPA-interacting protein that negatively regulates long-range resection; this inhibition is countered by export of HELB outside the nucleus in a cell cycle–specific manner during ongoing resection (89). BRCA1, along with its obligate binding partner BARD1, promotes end resection by stimulating the resection activity of BLM–DNA2 and increasing the DNA-binding efficiency of EXO1 (96, 97). 53BP1 antagonizes HR by associating with histone H4 methylated at lysine 20. During S/G_2_ phase, BRCA1–BARD1 recognizes the same histone residue contacted by 53BP1 to curb the antiresection activity of 53BP1 (90). Conversely, DYNLL1, a cytoplasmic motor protein, is recruited to the DSBs by 53BP1, and together they act as anti–end resection factors. DYNLL1 directly binds to MRE11 and interferes with its dimerization, thereby limiting MRE11’s nuclease activity (91, 92). Similarly to DYNLL1, the Shieldin complex localizes to DNA damage in a 53BP1-dependent manner, where it protects DNA from undergoing hyperresection (93). The Shieldin complex recruits the CST heterotrimeric complex, which further associates with POLα to fill in the resected DNA, thereby counteracting the end resection machinery (94, 95). The precise details of how these pro- and antiresection factors are coordinated remain to be determined.

## 3. MOLECULAR CHOREOGRAPHY: RAD51 AND PRESYNAPTIC FILAMENT FORMATION AND TARGET SEARCH

**Figure 2:**
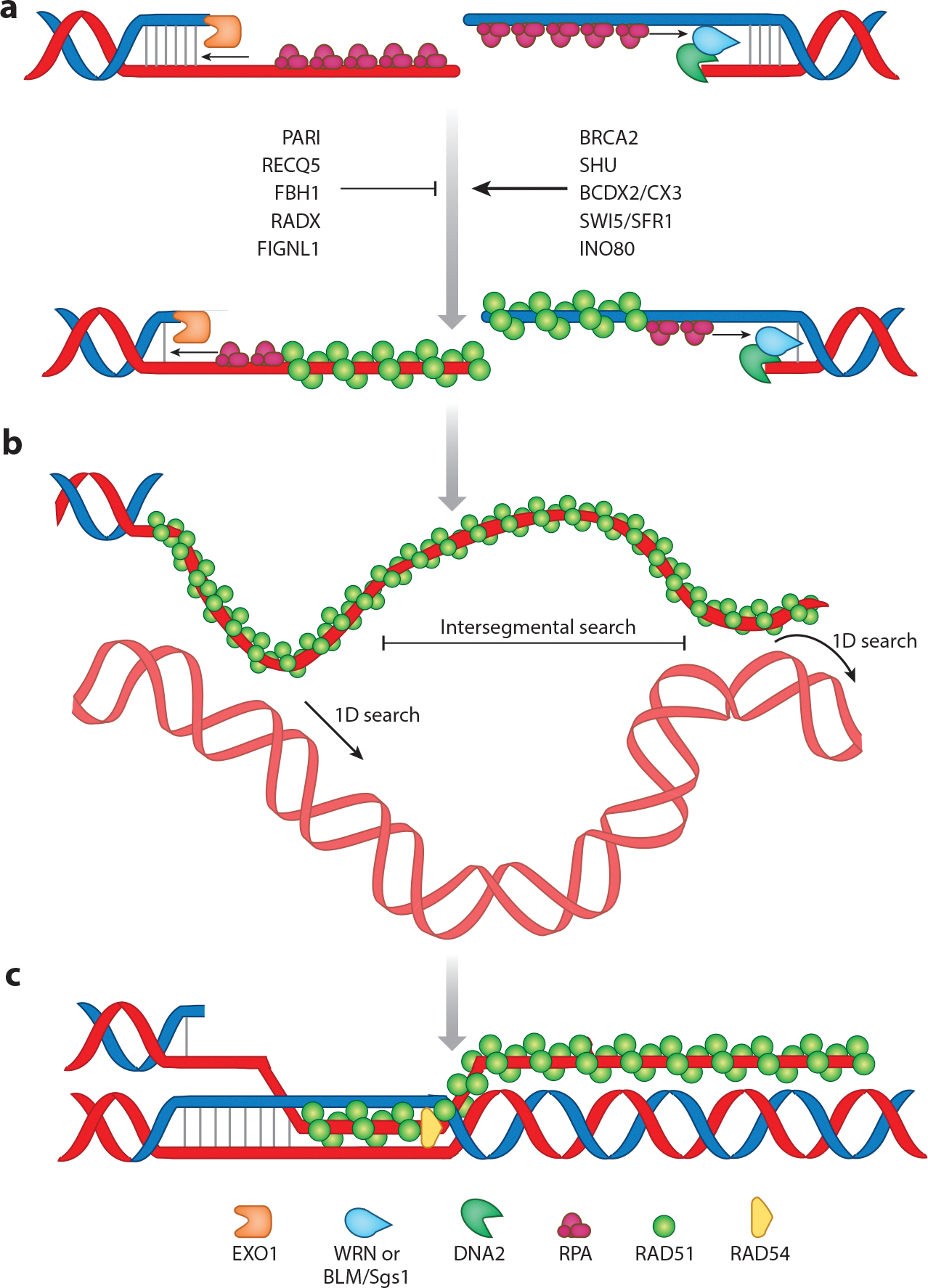
Presynaptic filament formation and homology search. (*a*) RAD51 replaces RPA on the single-stranded DNA leading to the formation of a RAD51–nucleoprotein filament, and this process is subject to both positive and negative regulatory factors. (*b*) The presynaptic filament can search for homology through a combination of diffusion-based processes (intersegmental transfer, 1D sliding). (*c*) Homology search is augmented by the motor protein RAD54, which can drive ATP-dependent translocation of the presynaptic filament along the double-stranded DNA. Abbreviations: 1D, one-dimensional; BLM, Bloom helicase; EXO1, exonuclease I; RPA, replication protein A; WRN,Werner helicase.

The hallmark of HR is the ability of the resected ssDNA to find, invade, and copy a homologous DNA sequence as a template for repair. This activity is carried out by Rad51, which binds to the long ssDNA generated during end resection (Figure 2). Rad51 was first identified in a genetic screen in *S. cerevisiae* as a mutant sensitive to ionizing radiation (98). Rad51 belongs to the RecA/RAD51 superfamily of proteins, which includes RecA and RadA (the bacterial and archaeal orthologs of Rad51, respectively) as well as a meiosis-specific homolog Dmc1 (see Section 5) (99, 100). Rad51 is highly conserved across eukaryotes, with mouse and human sequences being nearly 99% identical and yeast and humans sharing 67% identity (101). In mammals, homozygous deletion of *RAD51* results in embryonic lethality (102).

The structural features of Rad51 include an N-terminal lobe domain, an ATPase domain containing Walker A and Walker B motifs, two DNA-binding loops, and an interdomain linker (103, 104). Rad51 forms right-handed helical filaments on ssDNA in which the DNA is extended by ~50% relative to B-form DNA (105, 106). These nucleoprotein filaments are referred to as presynaptic complexes, and they are responsible for performing the homology search and strand-invasion reactions (106, 107). Nucleoprotein filament formation is a two-step reaction initiated by nucleation followed by filament extension (108, 109). Rad51 binds and hydrolyses ATP in a DNA-dependent manner. The observed *k*_cat_ of human RAD51 is 0.16 ATP/min and 0.05 ATP/min in the presence of ssDNA and dsDNA, respectively, compared to 25 ATP/min for bacterial RecA (110–112). Rad51 mutants that can bind ATP but are deficient in its hydrolysis can perform strand exchange in vitro, showing that ATP binding, but not hydrolysis, is required for activity. The ATPase activity of Rad51 is thought to be required for its dissociation from DNA (113, 114).

RPA–ssDNA is the initial intermediate that precedes formation of the presynaptic complex (Figure 2). The high abundance of RPA inside the cells, along with its high affinity toward ss-DNA (K_D_ ≈ 10^−10^ M, compared to K_D_ ≈ 10^−6^ M for Rad51), ensures that the resected ssDNA is more readily coated with RPA compared to Rad51 (115, 116). RPA protects ssDNA from degradation by the action of nucleases and is believed to aid Rad51 filament formation by removing the ssDNA secondary structures (117, 118). However, ssDNA-bound RPA also presents a barrier for Rad51 filament assembly. To overcome this barrier, Rad51 is aided by additional proteins, termed mediators, which positively influence Rad51’s replacement of RPA onto the ssDNA (Figure 2a). In contrast, there are also other proteins that negatively influence Rad51 filament formation to prevent aberrant or excessive recombination (Figure 2a).

Members of the Rad51/RecA family of recombinases have long been known to be ATP-dependent DNA-binding proteins that form extended helical filaments on DNA (103, 119, 120). Indeed, the bound DNA in these filaments was known to be extended by approximately 50% relative to an equivalent length of B-form DNA (105, 106, 121). However, for years, it was thought that the extension was uniform along the length of the DNA, and there was no reason to suspect otherwise. This view of the nucleoprotein architecture changed dramatically with the first published high-resolution structures of *Escherichia coli* RecA-bound DNA (122). These structures revealed that the extension of the DNA was not uniform but instead was organized into base triplets with near B-form configuration and one RecA monomer bound per triplet, and the phosphodiester bonds between the base triplets were extended to ~8 Å (120). As expected, this highly unusual architecture has now also been observed in high-resolution cryo-EM structures of eukaryotic Rad51/RecA family members, confirming that it reflects a fundamental property of the presynaptic complex (123). We are still trying to fully understand how this unusual DNA architecture impacts HR; one likely implication is that the necessary extension of a potential dsDNA target to match the length of the bound ssDNA would likely help to open up the duplex due to a loss of base stacking energy, thus assisting strand invasion.

### 3.1. Mediators of Rad51 Filament Formation

Rad51 filament formation is a rate-limiting step in HR, and there are a number of factors that positively regulate this process by accelerating Rad51 recruitment, by stabilizing the Rad51 filament, or by counteracting the negative regulators of Rad51 (Figure 2a) (100, 124, 125). Purified Rad51 can bind both ssDNA and dsDNA in vitro, and some translocases disrupt the assembly of Rad51 onto dsDNA to ensure the availability of free Rad51 to bind to ssDNA (126, 127).

Yeast Rad52 was one of the first proteins to be described as a mediator of Rad51. Rad52 physically interacts with Rad51 and stimulates its binding on RPA-coated ssDNA (128–130). Human cells also have RAD52, depletion of which results in a reduction in the number of RAD51 foci (131). However, in vitro experiments show that human RAD52, unlike yeast Rad52, cannot facilitate RAD51 nucleation on RPA-coated ssDNA; instead, this function is performed by BRCA2 (132). BRCA2 has eight BRC-repeat motifs and a C-terminal recombinase-binding region (CTRB, also known as TR2) that interacts with RAD51 (133, 134). Binding through the BRC repeats helps recruit RAD51 to the ssDNA and also inhibits the RAD51 ATPase activity, stabilizing the established filament (133, 135). CTRB-mediated binding also targets RAD51 to ssDNA and stimulates DNA strand exchange (136, 137). BRCA2, via its N-terminal domain, interacts with PALB2, which aides in recruitment to sites of DNA damage. PALB2 also interacts with BRCA1 and therefore allows formation of a BRCA1–BRCA2–PALB2 complex. Both BRCA1 and BRCA2 help recruit RAD51 to the DNA (138).

Rad51 paralogs aid the process of Rad51 filament formation. In *S. cerevisiae*, Rad55 and Rad57 are the two known Rad51 paralogs (139, 140). Yeast mutants lacking either Rad55 or Rad57 or both show defects in Rad51 assembly, deficient DSB repair by HR, and increased DNA-damage sensitivity (139, 141). Rad55 and Rad57 form a stable heterocomplex (142) and single-molecule studies have shown that Rad55–Rad57 transiently binds Rad51–ssDNA and stimulates Rad51 filament formation (143). In humans, the RAD51 paralogs that have been described include RAD51B, RAD51C, RAD51D, XRCC2, and XRCC3 (124, 144). The importance of mammalian RAD51 paralogs is underscored by the observation that null mutation of any of the RAD51 paralogs in mice results in embryonic lethality (145–147). The mammalian RAD51 paralogs form distinct complexes including BCDX2 (RAD51B, RAD51C, RAD51D, and XRCC2) and CX3 (RAD51C and XRCC3) (144, 148). Early insights into the ability of BCDX2 to act as the mediator of RAD51 filament formation came from studies in human cells showing that depletion of any of RAD51C, RAD51D, or XRCC2 led to an increase in DNA-damage sensitivity and a decrease in DNA damage–induced RAD51 foci (149). Recent biochemical and single-molecule analyses showed that the BCDX2 complex binds ssDNA but interacts only transiently with RAD51–ssDNA filaments (150). BCDX2 enhances RAD51 filament formation by facilitating the nucleation and extension of RAD51 loading on ssDNA (150, 151). Depletion of CX3 in human cells is dispensable for efficient RAD51 loading (152). However, CX3 positively regulates HR by acting at a later stage than BCDX2, potentially at the stage of Holliday junction (HJ) resolution, mechanistic details of which remain elusive (149).

Other factors like SWSAP1–SWS1 (the SHU complex), the SWI5–SFR1 complex, and the INO80 nucleosome-remodeling complex also stimulate RAD51 filament formation in humans. The SHU complex promotes RAD51 recruitment to DNA damage and also stabilizes the filament by counteracting the antirecombinase FIGNL1 (153). The SWI5–SFR1 complex directly interacts with RAD51, and depletion of SFR1 in human cells leads to fewer RAD51 foci (154). RAD51 recruitment to DNA damage and HR efficiency are reduced in the absence of INO80 (155). Although these protein complexes all promote RAD51 recruitment to damage sites, characterization of the exact mechanisms by which they function remains an active area of research.

### 3.2. Negative Regulators of the Presynaptic Complex

Most of the negative regulators of Rad51 are ATP-dependent DNA translocases that actively dismantle Rad51 filaments. Yeast Srs2, a UvrD family helicase, is one of the most well characterized negative regulators of the presynaptic filament. Srs2 is a 3′-to-5′ helicase and ssDNA translocase that physically interacts with Rad51 through its C-terminal region (156, 157). Biochemical and single-molecule studies have shown that Srs2 can processively remove Rad51 from ssDNA (158–160), and consistent with this negative regulatory role, *srs211* yeast cells show higher rates of recombination (161–163). Srs2 requires its translocase activity but not its helicase activity for removal of Rad51 from ssDNA (164). Rad51 binds weakly to ssDNA when in its ADP-bound state, and it has been postulated that Srs2 stimulates the ATP hydrolysis activity of Rad51, thereby promoting Rad51 filament disassembly (116, 160). The antirecombinase activity of Srs2 is counteracted by Rad54, Rdh54, and the Rad51 paralog complex Rad55–Rad57 (143, 165).

No clear Srs2 homolog has been found in vertebrates; however, several other helicases have been shown to fulfill a similar functional role. PARI is conserved among vertebrates and is known to contain a degenerate UvrD-like domain that does not possess helicase activity (166). Like Srs2, PARI-mediated disassembly of Rad51 is dependent on the ATPase activity of RAD51 (166). FBH1, a protein also belonging to the UvrD-type helicase family, is known to negatively regulate HR. FBH1-depleted human cells accumulate higher levels of RAD51 foci when the cells are presented with replication stress (167), and FBH1 can disassemble RAD51 filaments in vitro (168).

Other examples include RECQ5 and FIGNL1. RECQ5 is a RecQ-helicase family protein that contains a BRC-repeat variant through which it interacts with and disrupts RAD51 filament formation (169, 170). In contrast to yeast Srs2, human RECQ5 dismantles RAD51 filaments via a mechanism that is not dependent on the RAD51 ATPase activity (170). FIGNL1 is a AAA+ ATPase, knockdown of which results in persistent RAD51 foci after DNA damage (171). Supporting the in vivo findings, FIGNL1 promotes RAD51 dissociation in vitro from both dsDNA and ssDNA through its N-terminal domain (153). Since FIGNL1 can dissociate RAD51 from both dsDNA and ssDNA, it is hypothesized that FIGNL1 may exhibit disassembly activity by internally disrupting the RAD51 filaments rather than by translocation and successive interaction with available filament ends.

In addition to helicase-mediated negative regulatory factors, there are examples of proteins that downregulate RAD51 through other mechanisms. For example, the protein RADX is proposed to inhibit RAD51 filament elongation by binding and capping the ends of filaments (172, 173). Although negative regulators of RAD51 employ distinct mechanisms, further studies are required to paint a full picture of the interplay between these regulators and, more interestingly, their coordination with the mediators of RAD51.

### 3.3. Target Search and Strand Invasion

Presynaptic complexes must execute strand exchange resulting in the displacement of the original homologous strand and forming a structure called a displacement loop (D-loop). This raises the question of how exactly the presynaptic complex locates the correct region of homology. Initially, it was suggested that the homology search was carried out by probing the entire genome (174). However, estimates of the time required to complete this task did not fit a realistic time frame for efficient HR (175, 176). Numerous mechanisms may come into play to help accelerate the target search, including multiple modes of facilitated diffusion (e.g., intersegmental transfer and one-dimensional sliding) (177–179), searching for continuous short tracts of DNA rather than using a base pair–by–base pair search (180), and the involvement of ATP-dependent motor proteins to accelerate one-dimensional tracking of the presynaptic complex along a potential target duplex DNA (Figure 2b) (181). Studies have shown that bacterial RecA filaments can undergo intersegmental transfer, where a portion of the presynaptic filament is able to diffuse in three dimensions and establish multiple contacts with a DNA molecule while executing a hand-over-hand–type search (177). In this case, contacts with any nonhomologous DNA sequences are transient, and only once homology is recognized is a stable structure formed. RecA can also undergo one-dimensional diffusion of the presynaptic filament along the target DNA, which may accelerate strand invasion by reducing the dimensionality of the search process (178). RecA and Rad51 both appear to search for short tracts of homology, on the order of 8 bp in length, and very quickly reject any nonhomologous sequences shorter than this, thereby allowing the search process to focus only on longer tracts of homology that are most likely to be the correct target sequences (181, 182). In the case of yeast Rad51, the search can be further accelerated through the action of Rad54, which is an ATP-dependent motor protein that binds to the Rad51 presynaptic complex and moves along a potential dsDNA target while at the same time unwinding the stands to allow DNA pairing (Figure 2c) (181). Note that Rad54 is absent from bacterial systems, so this latter mechanism may be unique to eukaryotes (183). Nevertheless, it is important to recognize that these seemingly distinct mechanisms are not necessarily mutually exclusive, and they may all be taking place simultaneously to accelerate the search process. Moreover, these search mechanisms are very likely influenced by the overall organization of chromosomes within a cell. Specifically, the homology search is likely aided by the close spatial proximity of replicated sister chromatids, as HR generally takes place during the S and G_2_ phases of the cell cycle. Therefore, in most instances, a DSB is expected to already be in close spatial proximity to the template required for guiding repair (184, 185). However, repair can still take place between distal chromosomal locations, and in the absence of a repair template, the presynaptic complex can scan extensive regions while undergoing a frustrated search (186, 187), indicating that the search can expand beyond local regions (186, 188).

Once homology has been located, the presynaptic complex must pair its bound ssDNA with the homologous duplex to yield a D-loop intermediate (189, 190). In reconstituted settings, ATP hydrolysis is not required for the process of strand invasion to establish the paired D-loop intermediate (191–193). In addition, single-molecule studies have shown that these paired intermediates are stabilized in 3-nt increments (180, 194), which is consistent with the unusual structural organization of the bound DNA (123). D-loop intermediates can be extended by DNA polymerase from the 3′ end of the invading strand. These extended intermediates can be resolved by mechanistically distinct pathways yielding different recombination products.

## 4. MOLECULAR DECISIONS LEADING TO REPAIR PATHWAY CHOICE

DSB repair can proceed through gene conversion (GC), break-induced replication (BIR), single-strand annealing (SSA), or microhomology-mediated end joining (MMEJ). GC occurs when both the ends of the broken DNA share substantial homology to the donor DNA template, whereas BIR occurs when only one end of the broken DNA has homology present (195, 196). Breaks undergoing BIR generally occur because of defects in DNA replication such as replication fork breakage. A major difference between GC and BIR is that during GC only the regions around the DNA break are copied, whereas BIR involves synthesis of longer DNA tracts and can even extend all the way to the telomeres (197). SSA occurs when homologous repeat sequences are flanked at the DSB site. SSA requires end resection to reveal the flanking regions, followed by annealing of the sequences, gap filling, and ligation. MMEJ involves shorter tracts of sequence homology flanking the DSB sites and does not involve end resection. SSA and MMEJ are both RAD51-independent repair mechanisms, so our discussion focuses on GC and BIR. For more in-depth descriptions of SSA and MMEJ we refer readers to several recent reviews (198–201).

### 4.1. Gene Conversion

**Figure 3:**
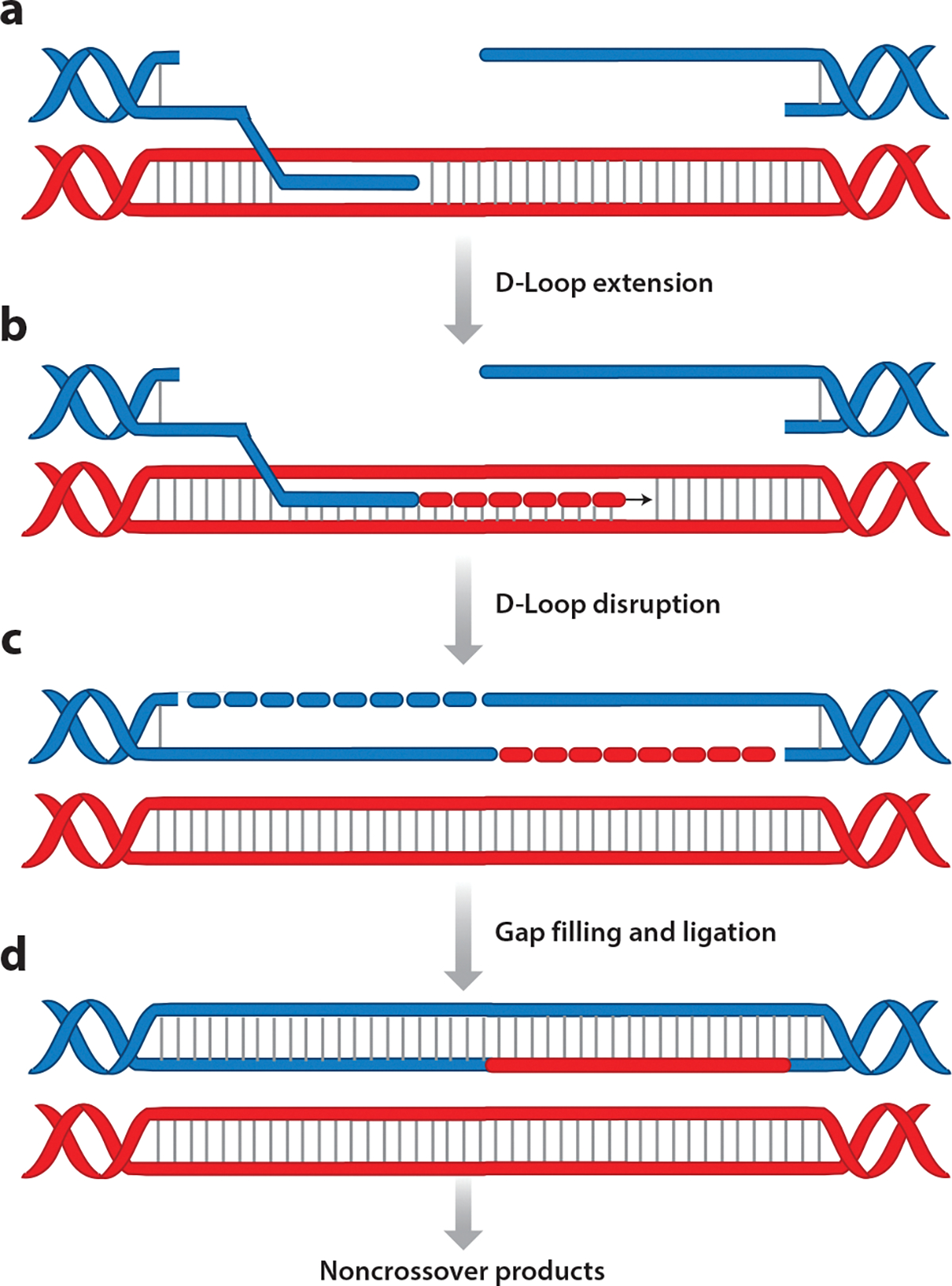
Synthesis-dependent strand annealing. (*a*) The single-stranded DNA formed after end resection is paired with the homologous template by Rad51 to yield a D-loop; note that Rad51 protein is not shown in the figure. (*b*) The invading 3′ end is extended by DNA synthesis. (*c*) The D-loop is then disrupted, allowing the newly synthesized end to be paired with the second DNA end. (*d*) The resulting intermediate is further processed by gap filling and ligation leading to the repair of the double-strand break. Abbreviation: D-loop, displacement loop.

**Figure 4:**
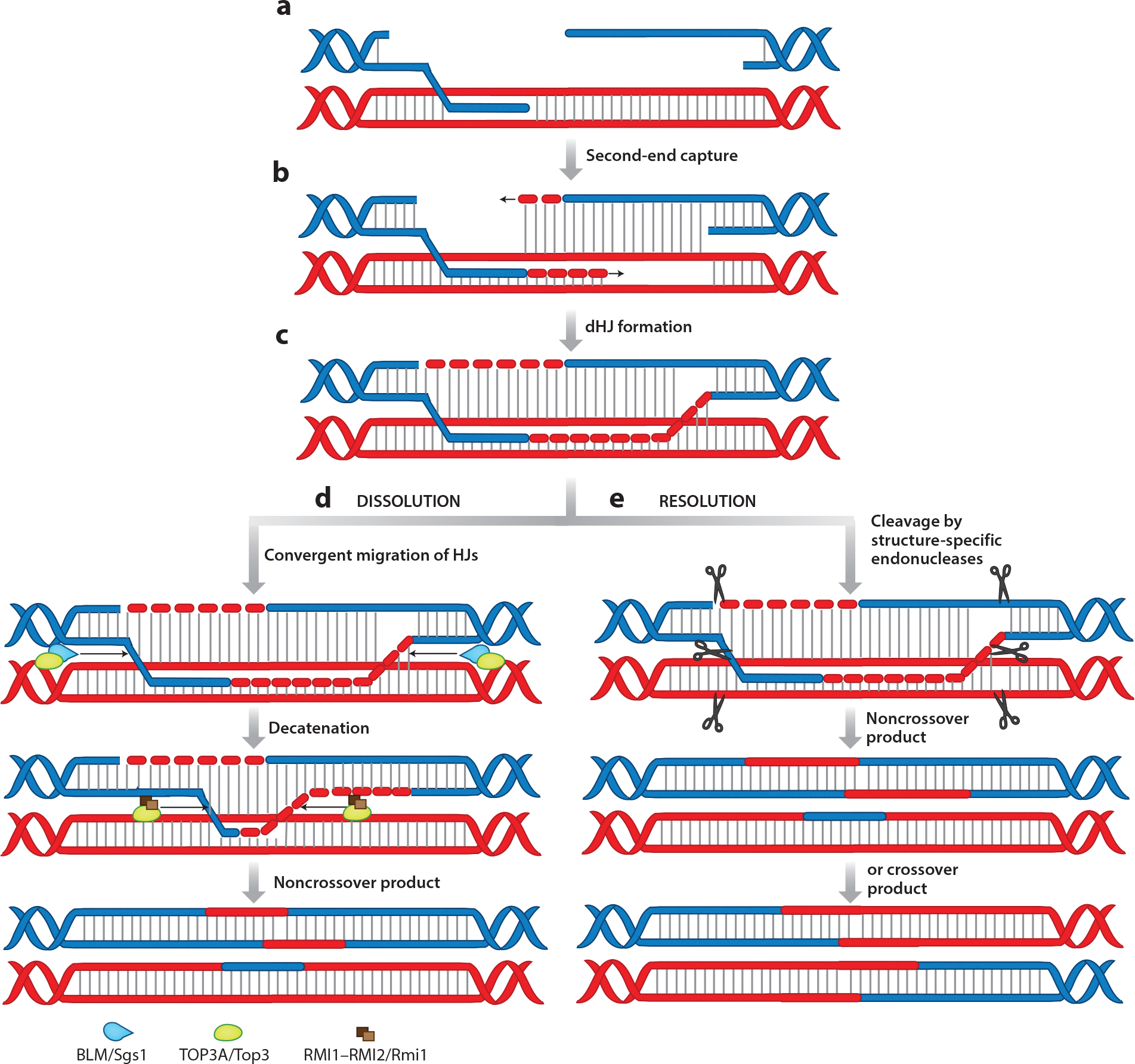
The DSBR pathway. (*a*) During DSBR, D-loop formation is followed by second-end capture. (*b*) DNA synthesis leads to the formation of a dHJ. (*c*) dHJs can be processed via dissolution and resolution. (*d*) In dissolution, helicases and topoisomerases coordinate to enable convergent migration of the two HJs, which are processed to give rise to noncrossover products. (*e*) In resolution, structure-specific endonucleases cleave the HJs to yield either a crossover or noncrossover product. Abbreviations: BLM, Bloom helicase; dHJ, double Holliday junction; D-loop, displacement loop; DSBR, double-strand break repair; HJ, Holliday junction.

GC can be divided into two subpathways: synthesis-dependent strand annealing (SDSA) (Figure 3) and canonical double-strand break repair (DSBR) (Figure 4). The repair outcome of SDSA is almost exclusively a noncrossover product. A signature of DSBR is the formation of a double Holliday junction (dHJ), which can be resolved into a crossover or a noncrossover product.

Strand invasion and D-loop extension is followed by the most critical step of the SDSA pathway: the dissociation of the extended D-loop from the heteroduplex DNA (Figure 3a,b,c). This allows annealing of the newly synthesized strand to the complementary sequence at the other DSB end. Strand annealing is followed by gap filling and DNA ligation to produce a noncrossover product (Figure 3d). Multiple proteins have been implicated in D-loop disruption during SDSA. Srs2, the yeast protein responsible for actively removing Rad51 from the ssDNA, can also disrupt D-loops in vitro (164, 202). Mammalian FANCM and its yeast counterpart Mph1 are other helicases that have been shown to dissociate D-loops, thus promoting SDSA (203, 204). The human protein RTEL1 can disrupt D-loops but not when they are bound by RAD51 (205).

In DSBR, the displaced strand can engage and anneal the ssDNA of the noninvading end in a process termed second-end capture (Figure 4a,b). Extension by DNA synthesis and strand joining via ligation can give rise to a classic dHJ structure (Figure 4c), which can be processed by either dissolution (Figure 4d) or resolution (Figure 4e). Dissolution of dHJs is coordinated by a multiprotein complex consisting of the BLM helicase (Sgs1 in yeast); the topoisomerase TOP3A; and two OB-fold-containing proteins, RMI1 and RMI2, together called the BTRR complex (Figure 4d). BLM drives convergent branch migration of the two junctions in an ATP-dependent manner, and TOP3A helps relieve topological stress (206, 207). Branch migration continues until a hemicatenane structure is obtained; this is then decatenated by TOP3A, which is stimulated by RMI1 and RMI2 (Figure 4d) (207–209). In resolution, the dHJ is cleaved by structure-specific resolvases, which specifically recognize and endonucleolytically cleave at or near the junction points on two diametrically opposing strands (Figure 4e). dHJ resolvases in humans include GEN1 and a complex of three different heterodimeric nucleases: MUS81–EME1, SLX1–SLX4, and XPF–ERCC1 (210, 211). Formation of the trinuclease complex allows them to function synergistically, and the trinuclease is more efficient than each of the nuclease complexes individually (212). Additionally, MSH2–MSH3, a heterodimer involved in MMR, stimulates the activity of the trinuclease resolvase complex via its interaction with SLX4 (213). Dissolution of the dHJ leads exclusively to noncrossovers and is considered a default pathway in somatic cells, whereas resolution can lead to both crossovers and noncrossovers (214, 215). Additionally, resolvases are activated later in the cell cycle than the BTRR complex, with trinuclease complex formation being enhanced only during prometaphase in a CDK- and PLK1-dependent manner, while GEN1 is excluded from the nucleus until M phase (211). For these reasons, resolvases are thought to be used as a last choice during DNA repair in somatic cells.

### 4.2. Break-Induced Replication

**Figure 5:**
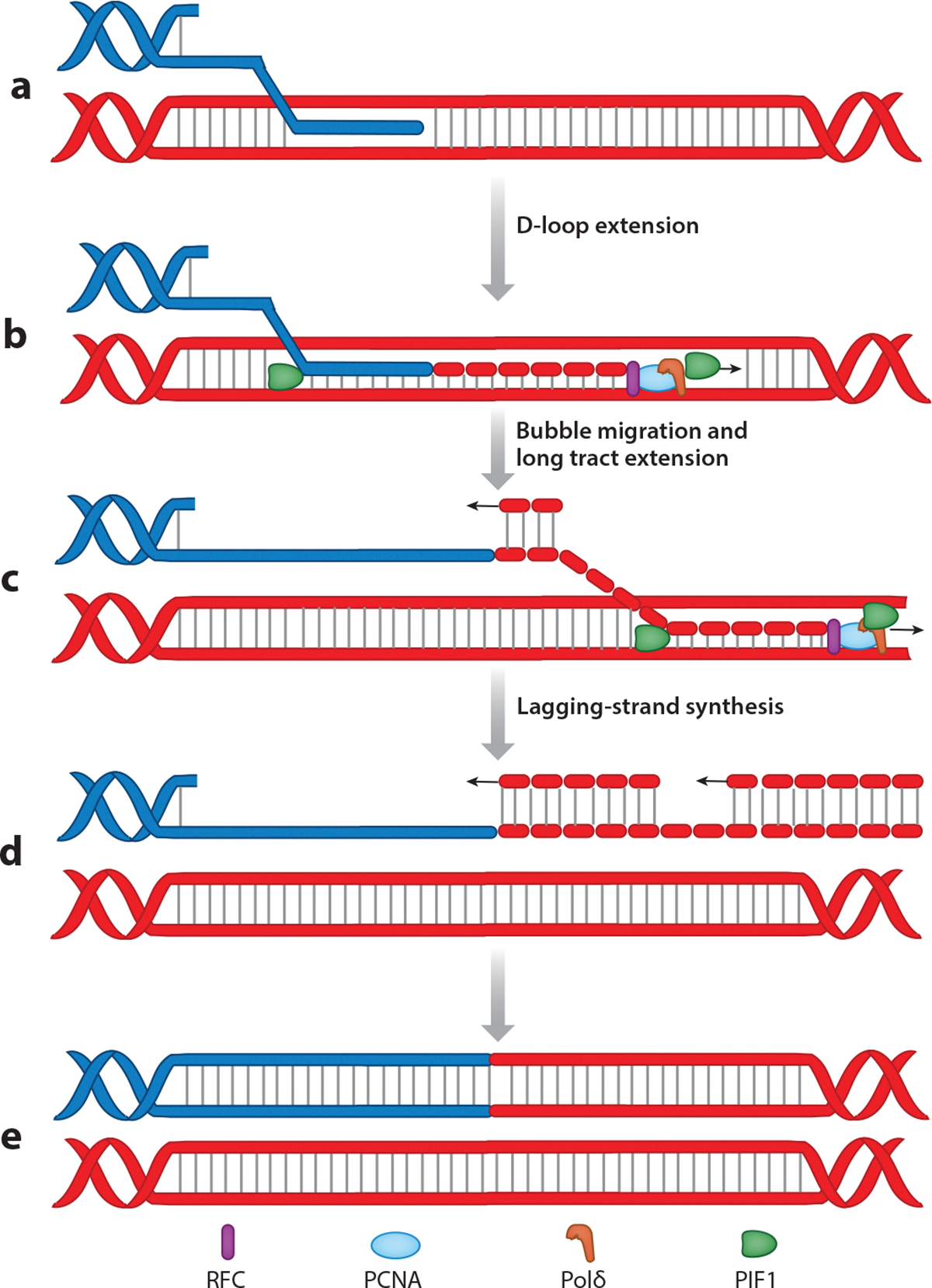
Break-induced replication (BIR). (*a*) BIR is initiated when there is homology present only to one end of the double-strand break (DSB). (*b*) Displacement loop (D-loop) formation takes place followed by extension. (*c*) PCNA, RFC, Polδ, and PIF1 coordinate bubble migration along with synthesis of long tracts of DNA. (*d*) The newly synthesized DNA is used as a template for replicating the lagging strand. (*e*) The newly formed double-stranded DNA is ligated, leading to the repair of the DSB.

BIR (or pathways having BIR-like characteristics) has been reported in several different organisms including bacteria, yeast, and mammals, as well as T4 bacteriophage (200, 216, 217). During BIR, DNA synthesis occurring at the 3′-OH end of the invaded strand is accompanied by migration of the unresolved HJ, resulting in displacement and accumulation of the newly synthesized ssDNA (Figure 5a–c) (218). The newly synthesized ssDNA is then used as the template for lagging-strand synthesis (Figure 5d,e) (218, 219).

An important decision the cell needs to make is whether to use GC or BIR. A recombination execution checkpoint is proposed to sense the presence or absence of two homologous regions at the break site, thereby helping with the decision (195). When only one region of homology is present, repair occurs through BIR, but when there are two homologous regions, cells undergo GC-mediated repair. Most of our knowledge of BIR comes from studies in budding yeast (220, 221). Multiple polymerases including Polα, Polδ, and Polε have been implicated in BIR in yeast. Polα plays a role in the initiation of leading-strand synthesis, and Polε promotes synthesis once BIR has already begun (222). Pol32, a subunit of Polδ, is essential for BIR and is also involved in strand displacement during D-loop migration. Polδ is considered the main polymerase required for leading-strand synthesis during BIR (222). The exact division of labor among these polymerases and the specific roles each play are still poorly understood. Pif1 is a helicase implicated in efficient long-tract BIR (223). In the absence of Pif1, BIR can still take place, but DNA synthesis does not occur over the same distances (224). The exact role of Pif1 in BIR has not been pinpointed yet. It is speculated that it can work either in front of the migrating D-loop to unwind the duplex DNA or alternatively behind the bubble to continuously unwind the newly synthesized DNA (Figure 5b,c). There is also evidence suggesting Pif1 may play a role in recruiting Polδ (223). Srs2 and Mph1 have also been shown to regulate BIR by dismantling toxic recombination intermediates formed behind the BIR bubble (225). BIR confers an elevated risk of accumulating mutations, which is generally attributed to the frequent dissociations and lower efficiency of the polymerases working within the context of a migrating D-loop compared to canonical DNA replication. Genetic studies suggest the existence of both Rad51-dependent and Rad51-independent BIR pathways, although mechanistic details of the Rad51-independent pathway remain enigmatic (200, 216, 217). Lastly, the main difference between BIR in yeast and mammalian cells is the length of the BIR track: In yeast, BIR can proceed for hundreds of kilobases, while in mammals, tract length rarely exceeds 4–5 kb (220, 221, 226).

## 5. MEIOTIC RECOMBINATION: AN ACT OF STAYING AWAY FROM SISTER

Meiosis is a specialized cell-division program characterized by two consecutive rounds of chromosomal segregation events preceded by a single round of DNA replication. During the first round, homologous chromosomes are segregated, while sister chromatids get separated during the second round. HR is used as a programmed event that leads to the formation of a physical linkage in the form of a crossover to ensure faithful segregation of the homologous chromosomes (227–229). In this section, we highlight features that distinguish meiotic recombination from the recombinational repair that takes place in somatic cells.

### 5.1. Initiation of Meiotic Recombination

**Figure 6:**
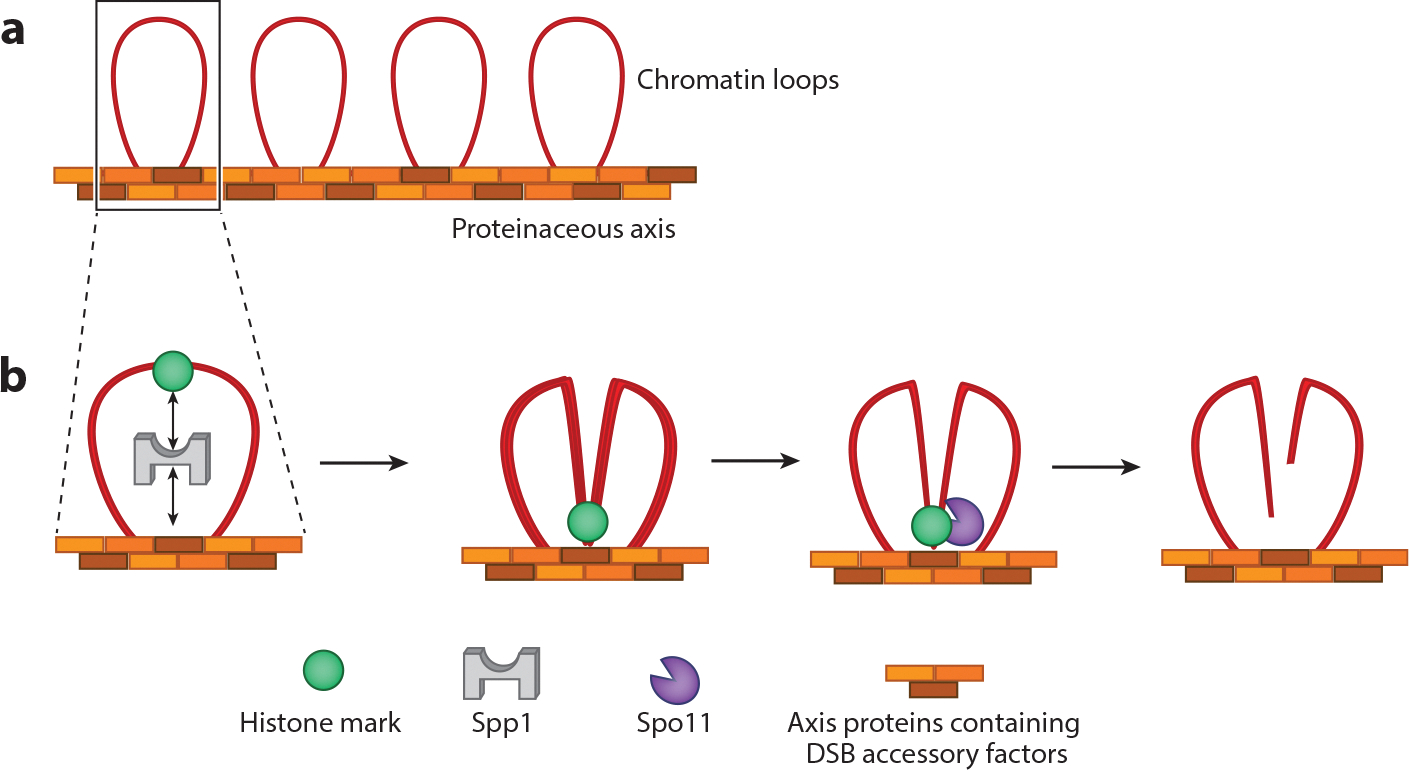
Meiotic DSB formation. (*a*) Meiotic chromosomes are organized into a series of chromatin loops that emanate from the proteinaceous axis, which consists of multiple proteins including those directly involved in catalyzing the DSBs. (*b*) Interactions between different proteins and DNA signatures help bring the DNA sequence on the loop into close proximity to the axis where DSBs are catalyzed. Abbreviation: DSB, double-strand break.

Meiotic recombination is initiated by deliberate introduction of DSBs. Cell cycle kinases and components of the DDR, along with proteins that maintain the meiotic chromosomal architecture, regulate the spatio-temporal distribution of these DSBs (230–232). In addition, the DSBs occur preferentially at DNA sites that are thought to be highly bendable, suggesting a requirement for a particular topological state rather than the presence of a strict sequence motif (233). In budding yeast, DSB formation is the result of the concerted efforts of at least ten different proteins. These proteins are divided into three different complexes: the core complex (Spo11, Ski8, Rec102, and Rec104), the RMM complex (Rec114, Mei4, and Mer2), and the MRX complex (234, 235). Key among these is Spo11, an evolutionarily conserved topoisomerase VI–like protein, which is responsible for cleaving the DNA (236, 237). Importantly, meiotic chromosomes are arranged along the proteinaceous chromosomal axis from which chromatin loops emanate (Figure 6a). Most of the abovementioned proteins are enriched along the chromosomal axis, whereas DSBs primarily occur within the distal chromatin loops (Figure 6a,b). This spatial conundrum is reconciled by a tethered loop-axis model in which chromatin loops are proposed to be captured by the DSB proteins present on the axis, allowing Spo11 to catalyze the break (Figure 6b) (238–240). In budding yeast, two important players in this process are Spp1, a histone reader, and Mer2, a subunit of the RMM complex. Spp1 recognizes histone marks on the chromatin loops and acts as a bridge by bringing the loop into close proximity to the chromosome axis via a direct interaction with Mer2 (Figure 6b) (241, 242). Mre11, a subunit of the MRX complex, is also involved in regulating DSB formation by directly interacting with Spo11 (243). Spo11 in turn promotes Mre11 recruitment, thus ensuring a direct connection between DSB formation and end resection (243). These DSB ends are then subjected to end resection in a manner similar to that used for repair in somatic cells.

In mammals, DSBs are also catalyzed by SPO11 with assistance from TOPOVIBL, a topoisomerase type IIB family protein, along with an auxiliary protein complex consisting of REC114, IHO1, MEI1, MEI4, and ANKRD31 (244–246). Meiotic DSBs have a nonrandom distribution with local chromosomal signatures like DNA sequence, chromatin accessibility, and histone marks influencing their location. PRDM9, via its histone methyltransferase activity and its ability to interact with HELLS, an SF2 family helicase, removes nucleosomes, ensuring maintenance of the open chromatin structure needed for efficient DSB formation (247, 248). Although the last decade has seen a major push toward using biochemical approaches to understand the functional intricacies of the proteins involved in DSB formation, the detailed molecular mechanisms governing the collaboration between these DSB proteins remain unclear. In the gametic cells in vertebrates, ssDNA generated by ongoing resection is coated with a heterodimeric complex of MEIOB–SPATA22 in addition to RPA (249, 250). Immunofluorescence experiments suggest that MEIOB and SPATA22 are also replaced by RAD51, similar to the replacement of RPA by RAD51 and its meiosis-specific homolog DMC1 during presynaptic complex assembly (251, 252). Functional differences and similarities between MEIOB–SPATA22 and RPA and their interplay with RAD51 and/or DMC1 are interesting topics that require further investigation.

### 5.2. Template Choice to Avoid the Sister

The key difference between recombination in meiotic cells and that in somatic cells is the use of a homologous chromosome as a donor for the repair of DSBs in meiotic cells instead of the sister chromosome, which is preferred in somatic cells. During budding yeast meiosis, an important factor that biases the repair toward interhomolog recombination is the differential regulation of the Rad51 and Dmc1 recombinases. Dmc1 is a meiosis-specific recombinase (253–255). Meiotic cells use Dmc1 for strand exchange instead of Rad51, whereas Rad51 plays a structural noncatalytic role (255, 256). Accessory factors play a pivotal role in generating the interhomolog bias. Hed1, a meiosis-specific Rad51 interacting protein, binds to Rad51 and prevents association of Rad51 with Rad54, thus downregulating the recombinase activity of Rad51 (182, 257). Rad54 and Hed1 are also the targets of activated Mek1 kinase activity. Rad54 phosphorylation by Mek1 reduces the ability of Rad54 to interact with Rad51, thus downregulating the strand exchange activity of Rad51 (258). Hed1 phosphorylation by Mek1 stabilizes the protein and thus enhances the Hed1-dependent downregulation of Rad51 (259). Conversely, the meiosis-specific proteins Hop2–Mnd1 and Sae3–Mei5 specifically stimulate the strand exchange activity of Dmc1 (260, 261). Thus, Rad51 and Dmc1 bind to different accessory proteins leading to downregulation of Rad51 and upregulation of Dmc1. Interestingly, although Dmc1 and Rad51 are closely related, Dmc1, unlike Rad51, is associated with tolerating mismatches in the base triplets during the process of homology search (180, 194, 262). This ability of Dmc1 may make it better equipped to form a stable heteroduplex DNA even with imperfectly matched recombination intermediates between homologous chromosomes.

The other major effector of the interhomolog bias in budding yeast is Mek1, an S/T kinase, along with proteins modulating the chromosomal architecture during the repair process (263, 264). Mek1 is the central kinase in the phosphokinase signaling cascade monitoring DSB formation and progression through meiosis (264, 265). Hop1, Red1, and Rec8 proteins are present at the axis of chromosomes through which chromatin loops emanate (228). The importance of axial proteins in establishing the interhomolog bias is underscored by the observation that in the absence of Red1, the interhomolog bias is reverted and DNA repair uses the sister chromosome as the template (266). Upon DSB formation, Hop1 is phosphorylated by the Mec1 and Tel1 kinases (ATR and ATM in mammals) (267). Phosphorylated Hop1 recruits Mek1 to the chromosomal axis, leading to the dimerization and activation of Mek1, which in turn phosphorylates a variety of targets (258, 268). One proposed mechanism by which a cell is able to distinguish sister chromosomes from homologous chromosomes involves activated Mek1 creating a local zone of inhibition blocking use of the spatially closer sister chromosome as a template, thus promoting interhomolog repair (269). How is this inhibition shut down? At the sites of DSB breaks, a zippering action of the synaptonemal complex brings the two homologs into close proximity (270, 271). Assembly of the synaptonemal complex at the interface of the homologous chromosomes leads to Hop1 removal from the axis by the action of a Pch2, a AAA+ ATPase (272–274). This change in the composition of axial components attenuates further DSB formation and the Mek1-dependent local inhibition of repair using the sister chromosome as the template (269).

### 5.3. Crossovers Over Noncrossovers in Meiosis

HR in somatic cells mainly yields noncrossover products, whereas HR during meiosis has a bias toward formation of crossovers (275). Crossovers mature into chiasmata, defined by a physical attachment between chromosome arms, which provide a physical linkage essential for faithful chromosome segregation during meiosis. Crossover interference, a process not well understood mechanistically, dictates that no two crossovers are placed in close proximity to each other and that crossovers are evenly spaced along the length of chromosomes. The suppression of crossovers is tightly regulated, as there needs to be at least one crossover per bivalent (276). Meiotic crossovers can be divided into class I and class II, with class I crossovers mostly preferred over class II crossovers during meiosis. Class I crossovers are subjected to crossover interference, whereas class II crossovers are not (277). In budding yeast, the formation of class I crossovers is primarily dependent on ZMM proteins (Zip1, Zip2, Zip3, Mer3, Msh4, Msh5, Spo16, and Spo22), Mlh1–Mlh3, Exo1, and the replication factors PCNA and RFC. ZMM proteins protect the recombination intermediates from the anticrossover activity of the Sgs1–Top3–Rml1 complex (278). The Msh4–Msh5 heterodimer binds to and stabilizes the HJ by forming a sliding clamp and embracing the DNA (279). Mlh1–Mlh3 also binds the HJ and has an endonuclease activity that is stimulated by Exo1, PCNA, and RFC (280–282). In contrast to class I crossovers, class II crossovers are resolved in a similar fashion to crossovers in somatic cells, with structure-specific endonucleases playing the main role (275). How is the choice between class I and class II crossovers made by the cells? There is growing evidence that ZMM proteins designate the recombination intermediates as future class I crossover sites, and they recruit factors responsible for channeling the recombination intermediates through class I crossover–dependent repair.

## 6. CONCLUDING REMARKS

HR is a fundamental process whose role spans crucial biological functions, including repairing damaged DNA and facilitating genetic diversity. The intricate, multistep mechanisms involved in HR highlight its complexity. Although considerable progress has been made toward understanding the individual steps involved, several mechanistic questions remain unanswered. DNA end resection is believed to be the step that commits DNA repair to HR. The minimum length of resected DNA required for this commitment, and the mechanism by which cells sense this length, still needs to be ascertained. Once cells have committed to HR, the processes that lead to the termination of end resection are also poorly understood. Homology search involves different dynamic search processes. Delineating the importance of these processes and understanding the role of the accessory factors therein are critical for a better understanding of HR. Mechanisms underlying the formation of the desired recombination product, especially that resulting in the bias toward crossover resolution in meiotic cells, remain poorly understood. Furthermore, the different steps taking place during DSB processing and repair are often depicted as separate stages occurring in sequential order; however, it is likely that these physiological processes take place concomitantly. Currently, very few details of how these reactions might be coordinated with one another have been described. In the context of human health, defects in HR are linked to various genetic disorders and cancers, underscoring the need for continued research to better understand its mechanisms and vulnerabilities. Advances in understanding HR have already led to the development of targeted cancer therapies like those involving PARP inhibitors. Continuing these efforts, for example, by performing synthetic lethality screens on cancer cells with defective HR pathways, offers significant potential for new advances toward developing novel cancer therapeutic targets.

## Figures and Tables

**Table 1:** Protein factors involved in homologous recombination

Human	*Saccharomyces cerevisiae* ^a^	Properties and function	Reference(s)
MRE11-RAD50-NBS1 (MRN complex)	Mre11-Rad50-Xrs2 (MRX complex)	Protein complex with nucleolytic and structural functions Recognizes DSBs Executes short-range DNA end resection	29–32
CtIP	Sae2	Modulates the activity of the MRN/MRX complex	51, 52
EXO1	Exo1	Nuclease Executes long-range DNA end resection	54, 58, 59
DNA2	Dna2	Nuclease (DNA2), helicase (BLM/WRN/Sgs1) Executes long-range DNA end resection	69–72
BLM	Sgs1		
WRN			
RPA70-RPA32-RPA14 (RPA complex)	RFA1-RFA2-RFA3 (RPA complex)	ssDNA-binding protein complex Protects resected DNA Prevents hyperresection	68
ATM	Tel1	Kinase Responsible for DNA damage response signaling	82, 83
ATR	Mec1	Kinase Responsible for DNA damage response signaling	85
HELB	NA	Negatively regulates long-range DNA end resection	89
53BP1	Rad9	Negatively regulates long-range DNA end resection	90
DYNLL1	NA		91, 92
SHLD1-SHLD2-SHLD3-REV7 (Shieldin complex)	NA	Protects DNA from undergoing hyperresection	93
CTC1-STN1-TEN1 (CST complex)	Cdc13-Stn1-Ten1 (CST complex)	ssDNA-binding protein complex Protects DNA from undergoing hyperresection	94, 95
BRCA1-BARD1	NA	Positively regulates long-range DNA end resection	96, 97
RAD51	Rad51	DNA-binding protein Performs strand exchange activity essential for HR	106, 107
BRCA2	Rad52	Positively regulates RAD51 filament formation	128–131
RAD51B-RAD51C-RAD51D-XRCC2 (BCDX2 complex)	Rad55-Rad57	RAD51 paralog complex Stimulates RAD51 filament formation	143, 149, 150
RAD51C, XRCC3 (CX3 complex)	NA	Positively regulates HR, potentially at the stage of Holliday junction resolution	149, 152
SWSAP1-SWS1 (SHU complex)	Csm2-Psy3-Shu1-Shu2 (Shu complex)	Stimulates RAD51 filament formation	153
INO80 complex	NA	Nucleosome remodeling complex Stimulates RAD51 filament formation	155
PARI	Srs2	Helicase, translocase Negatively regulates RAD51 filament formation	153, 158–160, 166, 168–170
FBH1			
RECQ5			
FIGNL1			
RADX	NA	Disrupts RAD51 filaments	172, 173
RAD54L	Rad54	Motor protein Accelerates the process of homology search	181, 182
FANCM	Mph1	Helicase Disrupts D-loops	203, 204
RTEL1	NA	Helicase Disrupts D-loops	205
BLM-TOP3A-RM11-RM12 (BTRR complex)	Sgs1-Top3-Rmi1 (STR comp1ex)	Involved in dissolution of double Holliday junctions	206, 207
MUS81-EME1	Mus81-Mms4	Nuclease	210–212, 214, 215
SLX1-SLX4	S1x1-S1x4	Involved in resolution of Holliday junctions	
XPF-ERCC1	Rad1-Rad10		
GEN1	Yen1		
MSH2-MSH3	Msh2-Msh3	Positively regulates resolution of double Holliday junctions	213
PIF1	Pif1	Helicase Efficient long-tract break-induced replication	223
SPO11	Spo11	Topoisomerase VI-like protein Responsible for programmed cleaving of the DNA during meiotic program	235–237
PRDM9	Spp1	Histone methyltransferase Required for efficient DSB formation during meiosis	247
HELLS	Irc5	Helicase Accessory protein required for efficient DSB formation	248
DMC1	Dmc1	DNA-binding protein Performs strand exchange activity essential for recombination during meiotic HR	253–256
NA	Hed1	Downregulates the recombinase activity of Rad51 during meiosis	182, 257
HOP2-MND1	Hop2-Mnd1	Stimulates the strand exchange activity of Dmc1 during meiosis	260
SWI5-MEI5	Sae3-Mei5	Stimulates the strand exchange activity of Dmc1 during meiosis	261
CHK2	Mek1	S/T kinase Central regulator of the phosphokinase signaling cascade monitoring the DSB formation and progression through meiosis	264, 265

Abbreviations: D-loop, displacement loop; DSB, double-strand break; HR, homologous recombination; NA, not applicable; ssDNA, single-stranded DNA.

a Homolog/functional equivalent.
